# Atypical AAA+ Subunit Packing Creates an Expanded Cavity for Disaggregation by the Protein-Remodeling Factor Hsp104

**DOI:** 10.1016/j.cell.2007.10.047

**Published:** 2007-12-28

**Authors:** Petra Wendler, James Shorter, Celia Plisson, Anil G. Cashikar, Susan Lindquist, Helen R. Saibil

**Affiliations:** 1 Department of Crystallography, Birkbeck College, Malet Street, London WC1E 7HX, UK; 2 Whitehead Institute for Biomedical Research, Nine Cambridge Center, Cambridge, MA 02142, USA; 3 Medical College of Georgia, 1410, Laney Walker Boulevard, Augusta, GA 30912, USA; 4 Department of Biochemistry and Biophysics, University of Pennsylvania School of Medicine, 805B Stellar-Chance Laboratories, 422 Curie Boulevard, Philadelphia, PA 19104-6059, USA

**Keywords:** CELLBIO, PROTEINS

## Abstract

Hsp104, a yeast protein-remodeling factor of the AAA+ (ATPases associated with various cellular activities) superfamily, and its homologs in bacteria and plants mediate cell recovery after severe stress by disaggregating denatured proteins through a poorly understood mechanism. Here, we present cryo-electron microscopy maps and domain fitting of Hsp104 hexamers, revealing an unusual arrangement of AAA+ modules with the prominent coiled-coil domain intercalated between the AAA+ domains. This packing results in a greatly expanded cavity, which is capped at either end by N- and C-terminal domains. The fitted structures as well as mutation of conserved coiled-coil arginines suggest that the coiled-coil domain plays a major role in the extraction of proteins from aggregates, providing conserved residues for key functions in ATP hydrolysis and potentially for substrate interaction. The large cavity could enable the uptake of polypeptide loops without a requirement for exposed N or C termini.

## Introduction

The 104 kDa protein-remodeling factor Hsp104 from *S. cerevisiae* disaggregates chemically or thermally denatured proteins in an ATP dependent manner, and cooperates with the Hsp70/Hsp40 chaperone system to refold the proteins to their native state (Glover and Lindquist, 1998). Aggregation was previously thought to be irreversible, but it is now clear that reactivation of aggregated proteins by Hsp104 is conserved in fungi (Glover and Lindquist, 1998), eubacteria (Mogk et al., 1999; Goloubinoff et al., 1999), and plants (Queitsch et al., 2000). After severe environmental stress, Hsp104 rescues essential proteins and increases cell survival by up to 10,000-fold (Sanchez and Lindquist, 1990; Sanchez et al., 1993). In yeast, Hsp104 also plays an essential role in the formation and inheritance of prions (Chernoff et al., 1995; Moriyama et al., 2000; Sondheimer and Lindquist, 2000; Shorter and Lindquist, 2004). Hsp104 disaggregation activity may have therapeutic potential as expression of Hsp104 reduces aberrant protein aggregation and increases longevity in rodents expressing mutant huntingtin (Vacher et al., 2005; Perrin et al., 2007).

Hsp104 is a member of the class1 Clp/Hsp100 subfamily of the AAA^+^ protein superfamily of ATPases (Schirmer et al., 1996). Functionally, the AAA^+^ superfamily is an extremely diverse group. However, the energy of ATP binding and hydrolysis is usually coupled to mechanical work such as unwinding or disassembling oligonucleotides or proteins. The active form of these proteins is typically a hexameric ring. Class1 Clp/Hsp100 proteins contain variable N- and C-terminal domains and two highly conserved AAA+ nucleotide binding domains (NBD1 and NBD2) separated by a variable middle region. X-ray crystal structures of subunits of the full-length, bacterial Clp/Hsp100 proteins ClpA (Guo et al., 2002) and ClpB (Lee et al., 2003) show that the two AAA^+^ domains are stacked head-to-tail. The structure of ClpB, the *T. thermophilus* homolog of Hsp104, reveals that the middle region is formed by two antiparallel coiled-coil motifs resembling a two-bladed propeller connected near the interface of NBD1 and NBD2 (Lee et al., 2003). In the crystal, the ClpB monomers do not form a hexamer, but rather assemble into a spiral containing three ClpB molecules in different conformations. Superposition of these structures indicates that the individual domains move as rigid bodies around hinge regions enabling a high mobility, in particular for the coiled-coil and N-terminal domains (Lee et al., 2003). Fitting of the ClpB crystal structure into cryo-EM reconstructions led to a hexameric model in which the coiled-coil domain protrudes from NBD1 on the outside of the complex (Lee et al., 2003, 2007).

Electron microscopy of negatively stained Hsp104 revealed a barrel-shaped hexamer of ∼155 Å diameter (Parsell et al., 1994). The hexameric state shows a higher ATPase rate than the monomer and is stabilized by high protein concentration, ADP or ATP, and low ionic strength (Schirmer et al., 2001, 1998, Hattendorf and Lindquist, 2002b). The major constitutive ATPase activity is driven by NBD1 (Schirmer et al., 1998; Hattendorf and Lindquist, 2002b), whereas nucleotide binding to NBD2 is crucial for oligomerisation (Parsell et al., 1994; Schirmer et al., 2001; Tkach and Glover, 2004). The ATPase activities of both domains are required for full protein remodeling activity and are modulated by allosteric communication within and between the two domains (Schirmer et al., 2001; Hattendorf and Lindquist, 2002b; Cashikar et al., 2002; Doyle et al., 2007). Communication between homotypic NBDs is thought to be mediated by arginine fingers (R334, R765 in Hsp104) that extend into the ATP binding site of adjacent subunits and participate in ATP hydrolysis (Karata et al., 1999).

The role of the coiled-coil region in Hsp104 activity remains enigmatic. This coiled-coil domain is found in Hsp100 proteins that are specialized for disaggregation. Indeed, deletion of this domain in ClpB abolishes protein remodeling but still allows some hexamerisation (Mogk et al., 2003), whereas single point mutations in a conserved 11-amino acid region of the coiled-coil domain of Hsp104 (helix L3) result in extremely diverse functional defects (Schirmer et al., 2004). A loss of function mutation in the equivalent region of Hsp101 can be restored by suppressor mutations in both NBD1 and the axial channel loops of the hexamer, indicating a functional link between the coiled coil and these regions (Lee et al., 2005) The coiled coil drives interdomain communication between NBD1 and NBD2 in Hsp104 (Cashikar et al., 2002) and acts as a regulatory device in ClpB by coupling translocation activity to DnaK chaperone activity (Haslberger et al., 2007). A mechanical model for ClpB suggests that ATP hydrolysis drives a ratchet movement that pulls apart protein aggregates by translocating individual polypeptides through its central pore (Weibezahn et al., 2004). This model is supported by a study showing that a modified *E. coli* ClpB protein (called BAP), containing the 26 amino acid ClpP binding motif of ClpA, unfolds aggregated substrates and delivers them to the ClpP protease for degradation (Weibezahn et al., 2004). However, there are other possible mechanisms for disaggregation that could, for example, include dissociation of the Hsp104/ClpB oligomer.

Conserved tyrosine residues in axial channel loops are strongly influenced by nucleotide binding to NBD2 and are crucial for disaggregation in *E. coli* and *S. cerevisiae* (Lum et al., 2004; Schlieker et al., 2004; Weibezahn et al., 2004). ClpB function also depends on the mobility of the coiled coil suggesting the coiled coil either adds a mechanical feature to the translocation process or supplies an additional activity to the complex (Lee et al., 2003; Watanabe et al., 2005). However, the mechanism of translocation and the role of the coiled coil, proposed to be on the exterior of the complex, are not explained by the current structural model (Lee et al., 2003, 2007).

In this study, we compare 3D structures of full-length and N-terminally truncated Hsp104 obtained by cryo-EM and single particle image analysis. Based on the cryo-EM maps of the complexes and a mutational study of conserved arginine residues, we show that the conformation of the Hsp104 hexamer in solution differs substantially from the previously published model of the ClpB hexamer. Rigid body domain docking of a Hsp104 homology model into the Hsp104 maps reveals an expanded AAA+ packing and coiled coil placement that has important implications for the mechanism of disaggregation activity in this broadly conserved protein family.

## Results

### Cryo-EM Maps of ΔN and Full-Length Hsp104 Hexamers Show an Ordered Ring of N Domains Capping a Large Chamber

**Figure 1:**
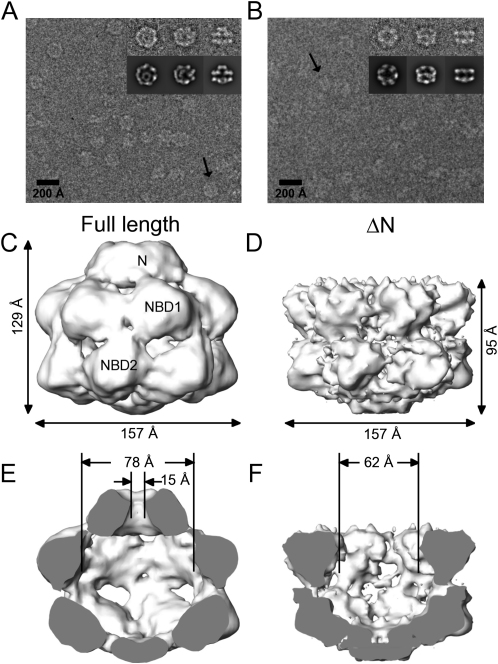
Cryo-EM Images and 3D Reconstructions of Hsp104 (A and B) Cryo-EM raw images of Hsp104^N728A^ (A) and Hsp104 ΔN (B) in the presence of ATPγS. Class averages containing 6–16 images of the final dataset, obtained by multivariate statistical analysis in IMAGIC, show characteristic views of the complexes (inset, upper row). Reprojections of the 3D structure in the Euler angle directions assigned to the class averages are included to judge the reconstruction (inset, lower row). The arrows indicate characteristic side views on the raw images. (C–F) Three-dimensional reconstruction of Hsp104^N728A^ and Hsp104 ΔN at 13 Å and 11 Å resolution as side views (C and D) and cut open side views (E and F). N, NBD1, and NBD2 indicate the assignment for the Hsp104 domains. Surface views show the density rendered at a threshold accounting for a molecular mass of 612 kDa (Hsp104^N728A^) and 500 kDa (Hsp104 ΔN).

Cryo-EM studies on Hsp100 proteins have so far not revealed any clear density for the N-terminal domains, suggesting that these domains are highly mobile in Hsp100 proteins and undergo movements of at least 30 Å (Lee et al., 2003; Ishikawa et al., 2004). To examine the layout of Hsp104, we compared the hexameric structures of full-length Hsp104 (Hsp104^N728A^) with an N-terminal deletion mutant (Hsp104 ΔN) (Figure 1). All data were collected in presence of the ATP analog ATPγS to promote a stable oligomeric assembly. The sensor-1 mutation in NBD2 has no effect on nucleotide binding but reduces ATP hydrolysis by Hsp104 at low protein and ATP concentrations, and elicits several protein-remodeling activities (Hattendorf and Lindquist, 2002b; Doyle et al., 2007). Analysis of Hsp104 ΔN top views revealed 6-fold symmetry (see Figure S1A available online) and this was used for single particle reconstruction of both maps. Refinement of the maps yielded structures at 11 and 13 Å resolution for Hsp104 ΔN and Hsp104^N728A^ respectively.

Hsp104^N728A^ forms a three-layered hexameric complex. A two-tiered ring structure capped by a smaller ring on one surface is evident in the raw images and more distinct in the class averages (Figure 1A). On the other hand, Hsp104 ΔN always appears as a double-layered ring structure in side view (Figure 1B). In both 3D reconstructions, individual domains in the upper and lower rings are well defined and can be attributed to the two NBDs. At the threshold shown, the small ring of density in the map of full-length Hsp104 accounts for the expected mass of 6 Hsp104 N termini (110 kDa). Since we can assign the small ring to the N termini, the middle and the lower ring of this map must be NBD1 and NBD2 respectively.

The 3D reconstructions of full-length and ΔN Hsp104 have a diameter of 157 Å and a height of 129 Å and 95 Å respectively (Figures 1C and 1D). In comparison to related cryo-EM structures, Hsp104 is ∼30 Å wider than ClpA (Ishikawa et al., 2004) and has the same outer diameter as the NBD2 ring of *T. thermophilus* ClpB AMPPNP (Lee et al., 2007). The measured diameter is consistent with earlier negative stain results on Hsp104 (Parsell et al., 1994). On the inside, the hexamer encloses a cavity of 73 Å in height and up to 78 Å in diameter at NBD1 (Figure 1E). The entrance to this cavity is restricted by a 15 Å channel in the N-terminal ring whereas the exit at the C-terminal end is either closed or smaller than the resolution limit.

Even though the outside diameter of the Hsp104 ΔN hexamer matches that of the full-length complex, the cavity in NBD1 is 16 Å narrower for the N-terminal deletion mutant (Figure 1F). This is mainly due to a rearrangement in the NBD1 domain of Hsp104 ΔN, as can be seen in an overlay of the maps (Figure S2). Lacking the ring formed by the N termini, NBD1 tilts into the cavity and the contact area between neighboring NBD1 domains becomes smaller than for these domains in the full-length protein. However, the only phenotype observed so far with the N-terminal deletion is an inability to cure cells of the [*PSI*^+^] prion (a self-templating Sup35 amyloid) when overexpressed (Hung and Masison, 2006).

### Domain Fitting Reveals that the Coiled-Coil Domain Is Intercalated between Subunits

**Figure 2:**
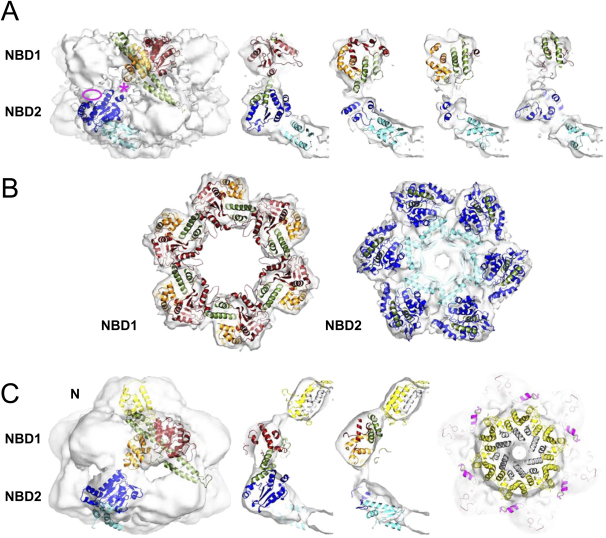
Docking of the Hsp104 Homology Model into the Cryo-EM Maps (A) Rigid body domain fitting into the Hsp104 ΔN map. Domains and subdomains are color coded as follows: NBD1, red/orange; NBD2, blue/cyan; coiled coil, green. The ATP binding pocket is located at the interface between the NBD subdomains (red/orange, blue/cyan). Left: front view of EM map with one monomer fitted. The connection between NBD1 and NBD2 (^∗^) and density in the NBD2 layer not filled by the docked structure (ellipse) are indicated. Right: 20 Å slices through the side view, in which the fitted hexamer is rotated anticlockwise through a 60° spherical segment. (B) Cross sections through the fitted NBD1 and NBD2 rings of Hsp104 ΔN. Color code is as in (A). (C) Rigid body fit into the Hsp104^N728A^ map. Left: front view with same color code as in (A) and N termini depicted in yellow. Middle: 20 Å slices through the side view. The N-terminal region missing from the Hsp104 homology model is filled in by equivalent ClpB residues and colored in gray. Right: cross-section through the fitted N-terminal ring. Helix L3 in the coiled coil is colored in magenta.

The assignment of N and NBD layers in both maps is clear from the preceding comparison and the alignment of the ΔN map to the full-length map (see Methods). Next, we determined the hand of the full-length Hsp104 map by tilt experiments (Figure S3). The domain arrangement of the maps is incompatible with the ClpB crystal structure (Lee et al., 2003), so that an Hsp104 homology model was separated into N, NBD1, NBD2 and coiled-coil domains for rigid body fitting. Since the ΔN structure was better resolved, it was used to establish the domain layout (Figure 2A and 2B). Automated fitting of NBD2 places the connection to NBD1 at the interface between the two layers (Figure 2A, ^∗^). The short hinge region between the AAA+ domains (10 amino acids) requires NBD1 to be placed to the upper right of NBD2. Fitting the boomerang-shaped cross section of NBD1 (Figure 2B) into the round density leaves an empty pocket on the cavity-facing side of NBD1, which accommodates the helix L3-bearing end of the coiled-coil motif well in length and diameter. Consequently, the helix L1/L2-bearing end of the coiled-coil domain passes through the stronger of the two density connections between the AAA+ domains and occupies the unfilled density at the outer top surface of NBD2. Although this fit is not accurate in fine detail and requires some refolding of the linker regions between NBD1 and the coiled coil, it is compatible with all the interdomain connections and clearly provides a good fit to the EM density. The coiled coil follows a similar path in the full-length structure, but it tilts to follow the more outward rotated orientation of NBD1.

Since the N termini of Hsp100 proteins share little sequence identity, homology modeling for Hsp104 residues 1-48 failed. Filling in with the ClpB X-ray structure, the N termini fit the density of the full-length Hsp104 EM map well and show tight packing in the ring (Figure 2C). The N-terminal ring of density in the full-length Hsp104 map is linked with NBD1 by two connections, the stronger of which is at the position where the helix L3 end of the coiled-coil domain emerges, suggesting that the tip of the coiled coil interacts with the N-terminal ring. It should be noted that the flexible connection to the N domain would also allow it to occupy the position to the right of the one shown. In that case, its interaction with the coiled coil would be inter- rather than intra-subunit. Hsp104 also contains a 40 amino acid, highly acidic C-terminal domain that is lacking in the bacterial ClpB variants and in the homology model. Fitting of NBD2 in the EM maps leaves vacant density in the bottom layer with the correct volume to account for the 6 C termini (Figure 2B).

**Figure 3:**
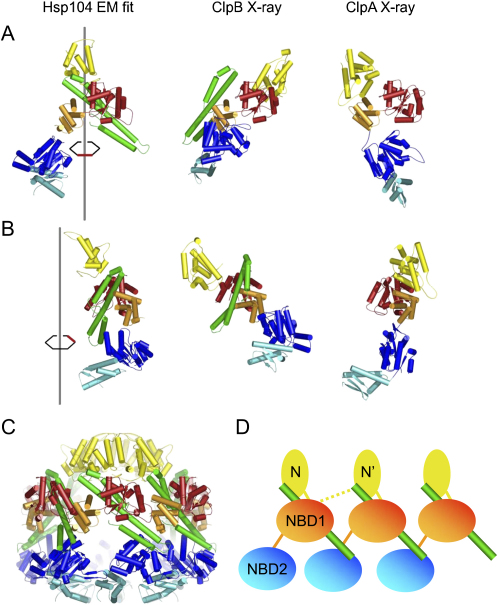
Comparison of Subunits from the ClpB and ClpA Crystal Structures with the Hsp104 Hexamer Model Comparison of subunits from the ClpB (1QVR_A, Lee et al., 2003) and ClpA (1R6B, Xia et al., 2004) crystal structures with the Hsp104 hexamer model. (A) Front view of the three structures aligned by superimposition of the Cα's in NBD1. The position of Hsp104 NBD1 along the rotational axis of the Hsp104 hexamer is indicated in red. Color code is as in Figure 2. (B) Same as in (A), but with 120° anticlockwise rotation of the monomers. (C) Hexameric assembly of Hsp104 homology model derived from the fit into the cryo EM maps. (D) Cartoon of subunit arrangement in Hsp104 oligomer. NBD and N domains are labeled and color coded as in (A). The length of the flexible hinge between N and NBD1 allows either N or N' to connect to NBD1.

When the monomeric Hsp100 structures from the crystal and the EM fits are superimposed through the Cα atoms of NBD1, the most significant difference is the position of the coiled-coil domain (Figure 3). In the EM conformation, it is located on the inside of NBD1, forming a close contact with this domain, whereas the crystal conformation positions it on the outside without major contacts to the rest of the molecule (Figure 3A). Nevertheless, when viewed from the side (Figure 3B), the relative positions of the coiled coil and NBD1 are similar. Thus, the EM conformation can be generated from the crystal conformation by rotating the coiled coil ∼90° clockwise around helix L2 and ∼90° anticlockwise around the hinge connection to NBD1. The orientation of NBD2 and the N-terminal domain in the fit to the EM map differ from those of ClpB and ClpA subunit crystal structures, emphasizing the mobility around the hinge points. In our structure, the coiled coil contacts an N domain at the L3/L4 end, interacts extensively with NBD1 of the same subunit and contacts NBD2 of the adjacent subunit through the L1/L2 end, providing a first glimpse of the structural basis for the complex allosteric interactions in this protein family (Figures 3C and 3D).

### Hsp104 Forms a Highly Expanded Hexamer Compared to p97 and Other AAA Complexes

Hexameric crystal structures of p97, HslU and the LTag of SV40 show that the oligomeric packing of AAA domains is conserved (DeLaBarre and Brunger, 2003; Huyton et al., 2003; Bochtler et al., 2000; Gai et al., 2004). These follow the principle that a conserved arginine residue of one AAA^+^ domain points into the ATP binding groove of the adjacent domain to facilitate ATP hydrolysis. Since the overall dimensions of the structurally conserved AAA^+^ domains are very similar, the maximum diameter of an AAA^+^ hexamer assembled with this packing is limited. We found that none of the AAA rings of the above-mentioned hexameric protein complexes are wider than 130 Å in any nucleotide state observed. Accordingly the maximum pore size formed by the complexes is ∼25 Å.

**Figure 4:**
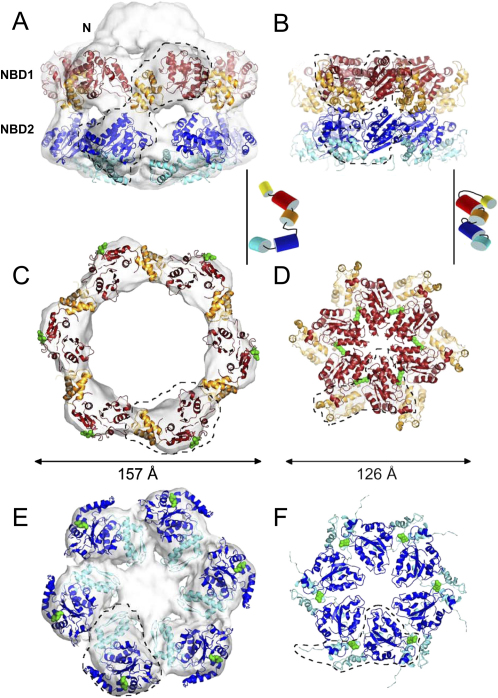
Comparison of Domain Packing in Hsp104^N728A^ and p97 Comparison of domain packing in Hsp104^N728A^ and p97 (1OZ4, DeLaBarre and Brunger, 2003). (A–F) Only the AAA+ domains of Hsp104^N728A^ (A, C, E) and p97 (B, D, F) are shown. The color code is as in Figure 2. Front views (A and B) and top views of the D1 (C and D) and D2 (E and F) rings are shown. Predicted arginine fingers are shown as green spheres. The position of one monomer within the hexamer is indicated by a dashed outline. The cartoons between top and middle rows indicate the positioning of individual domains relative to the rotational axis of the hexamer (black line).

In Figure 4, the EM map of Hsp104 N728A is compared to the crystal structure of the double AAA domain hexamer p97 (DeLaBarre and Brunger, 2003). The hexameric arrangement of the AAA^+^ domains in Hsp104 differs substantially from that of p97. With an outer diameter of nearly 160 Å and a pore size of up to 78 Å in the NBD1 ring, the packing of the AAA modules is significantly expanded compared to that in p97. Furthermore, we observe well-separated domains with little contact to adjacent domains, whereas individual AAA^+^ domains in p97 interlock with each other and share large interaction surfaces. When viewed down the 6-fold axis from the N-terminal surface, Hsp104 AAA+ domains are rotated clockwise relative to p97's by ∼45° in NBD1 (Figures 4C and 4D) and by ∼90° in NBD2. (Figures 4E and 4F). The predicted arginine fingers (green) in Hsp104 (R334, R765) are thereby located on the outside of the molecule and cannot contact the ATP binding site of the adjacent subunit in either of the AAA^+^ rings. This arrangement is distinct from crystal structures of AAA+ rings, which show interlocked packing in all nucleotide states. The fact that we detect a much larger pore size as well as an unusual arrangement of the AAA^+^ domains implies that the functional assembly of Hsp104 in solution differs from those of other AAA^+^ proteins so far described.

Furthermore, the presence of the coiled coil in the unfilled density on the inside of NBD1 blocks access to its ATP binding site and the arginine finger interaction between adjacent subunits (Figure 2B). The helix L1/L2 end of the coiled coil contacts NBD2 in the vicinity of R765 in our fit. Since R334 and R765 are poorly positioned to act as classical arginine fingers, we searched for other conserved arginines that might perform this function.

### Mutations of Conserved Arginines in the Coiled Coil Have Major Effects on Hsp104 Activity

**Figure 5:**
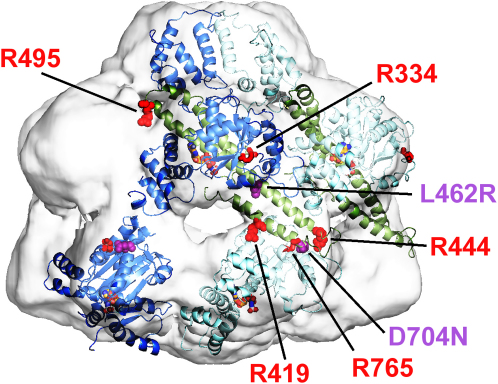
Positions of Key Residues in the Hsp104 Hexamer Model Two Hsp104 monomers, colored in dark and light blue, are fitted into the EM map. The coiled-coil domain is colored green in both monomers. Conserved arginine residues R419, R444, and R495 are shown as red spheres. Predicted arginine fingers R334 and R765 are shown as red sticks. L462R and D794N mutations are shown as magenta spheres. ATP is shown in ball and stick representation.

A sequence alignment of 26 Hsp100 proteins revealed the presence of three highly conserved arginine residues within the coiled-coil domain: R419, R444, and R495 (Figure S4). Arginine 419 is located on Helix L1 of the coiled coil and in our model could potentially act as an arginine finger for NBD1. Arginine 495 and R444 are positioned at opposite ends of Helix L2 with R444 contacting NBD2 of the neighboring subunit (Figure 5). To investigate potential roles of these arginine residues in ATP hydrolysis we mutated each to methionine and compared them with effects of mutating the predicted arginine fingers R334 (NBD1) and R765 (NBD2) (Neuwald et al., 1999).

**Figure 6:**
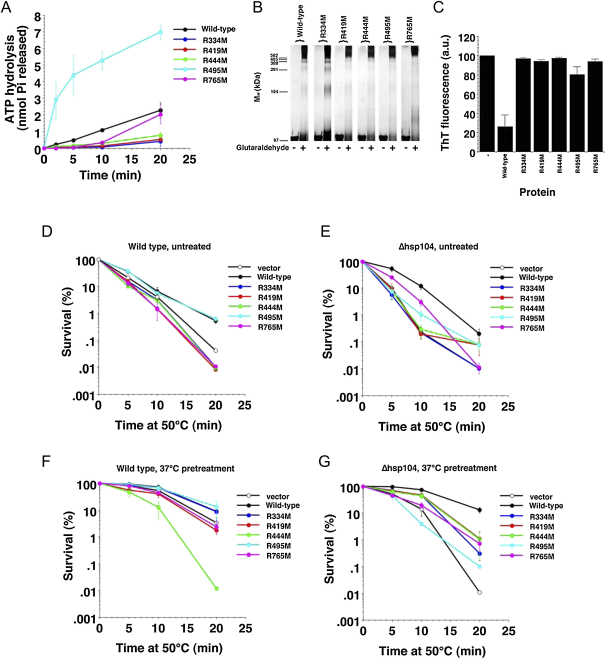
Biochemical and Functional Consequences of Mutating Conserved Arginine 334, 419, 444, 495, or 765 in Hsp104 (A) Wild-type or mutant Hsp104 proteins (0.2 μM) were incubated for 0–20 min at 25°C with ATP (1 mM). At various times the amount of ATP hydrolysis was determined. Values represent the mean ± SD (n = 5). (B) Wild-type (wt) or mutant Hsp104 proteins (0.2 μM) in the presence of ATP (1 mM) were either crosslinked with 0.1% glutaraldehyde for 10 min or left untreated. (C) NM fibers (2.5 μM monomer) were incubated with wild-type or the indicated mutant Hsp104 (2 μM) plus ATP (10 mM) for 60 min at 25°C. Disassembly was monitored by thioflavin T (ThT) fluorescence. Values represent the mean ± SD (n = 4). (D–G) Wild-type (D and F) or Δ*hsp104* (E and G) cells harboring the indicated plasmid were grown to mid-log phase (5 × 10^6^ cells/ml) in SD-ura liquid. Prior to the 50°C heat treatment, matched cultures were either maintained at 30°C (D and E) or preincubated at 37°C for 30 min (F and G). Following treatment at 50°C for 0–20 min cells were transferred to ice, diluted in ice-cold SD-ura, and immediately plated on SD-ura. Values represent the mean ± SD (n = 3).

Since the arginine finger catalyzes ATP hydrolysis in the adjacent subunit by stabilizing the developing negative charge of the transition state, we expect ATPase activity to be reduced when this arginine is mutated to the non-polar amino acid methionine. Indeed, the previously predicted NBD1 arginine finger mutant Hsp104^R334M^ as well as the coiled-coil domain mutants Hsp104^R419M^ and Hsp104^R444M^ each reduced the ATPase activity to ∼20%–30% of wild-type after 20 min at 25°C (Figure 6A). The predicted NBD2 arginine finger mutant, Hsp104^R765M^, initially displayed reduced ATPase activity, but by the end of the reaction ATPase activity approached wild-type levels. Unexpectedly, Hsp104^R495M^ had ∼3-fold higher ATPase activity than wild-type Hsp104, and the initial rate was more than 10-fold higher than wild-type. Thus, all the conserved arginines affected ATP hydrolysis in some manner.

In the AAA+ arrangements so far described, oligomerisation is crucial for the arginine finger contact between adjacent subunits to be made. Therefore, we used glutaraldehyde crosslinking to examine whether the various Hsp104 mutants formed hexamers. In the absence of nucleotide, wild-type Hsp104 and most of the mutant proteins failed to hexamerize. The single exception was Hsp104^R765M^, which surprisingly, could assemble into oligomers without nucleotide (Figure S5), perhaps through an intersubunit interaction between the coiled coil and the neighboring NDB2.

In the presence of ATP, all the mutants except Hsp104^R334M^ assembled into oligomers just as well as wild-type Hsp104 (Figure 6B). Therefore, defective hexamer assembly cannot explain their aberrant ATPase activity. Hsp104^R334M^ formed hexamers, but also yielded a significant population of monomers and smaller Hsp104 oligomers, indicating that this mutant is less assembly competent (Figure 6B). Thus R334 is the first NBD1 residue known to affect Hsp104 hexamer assembly, since oligomer formation has so far been attributed to NBD2 (Parsell et al., 1994; Schirmer et al., 2001).

Next, we tested the disaggregation activity of the arginine mutants. Hsp104 controls the formation and propagation of the yeast prion [*PSI^+^*] (Chernoff et al., 1995), which is comprised of self-perpetuating amyloid fibers generated by the prion domain, NM, of Sup35. Hsp104 rapidly solubilizes the unusually stable β sheet-rich conformation of NM fibers (Figure 6C). By contrast, all of the arginine mutants were defective in NM fiber disassembly. Of these, Hsp104^R495M^ was the least compromised, with ∼30% of wild-type activity, but Hsp104^R334M^, Hsp104^R419M^, Hsp104^R444M^ and Hsp104^R765M^ reached only 5%–10% of wild-type disaggregation levels.

Finally, we tested the importance of the conserved arginines for thermotolerance in vivo. In untreated wild-type cells, cell survival was moderately reduced by Hsp104^R334M^, Hsp104^R419M^, Hsp104^R444M^ and Hsp104^R765M^ relative to the vector control (Figure 6D), suggesting that these arginine mutants disrupted the thermotolerance function of endogenous Hsp104. In contrast, expression of Hsp104^R495M^ or wild-type Hsp104 increased cell survival after 20 min at 50°C by an order of magnitude. In untreated Δ*hsp104* cells, wild-type Hsp104 increased survival 20-50-fold compared to the vector control (Figure 6E). Hsp104^R334M^ conferred no more cell survival than the vector control. The other arginine mutants were all deficient in basal thermotolerance.

In preconditioned wild-type cells, only Hsp104^R444M^ greatly interfered with the thermotolerance function of endogenous Hsp104 (Figure 6F). This suggests that heat-inducible factors are able to buffer the otherwise inhibitory effects of Hsp104^R334M^, Hsp104^R419M^ and Hsp104^R765M^. In preconditioned Δ*hsp104* cells, wild-type Hsp104 increased cell survival ∼1100-fold over the vector control (Figure 6G). However, Hsp104^R419M^, Hsp104^R444M^, and Hsp104^R765M^ only increased survival by ∼60-100-fold. Hsp104^R334M^ and Hsp104^R495M^ conferred even less survival. Thus, all of the arginine mutants display greatly reduced induced thermotolerance.

## Discussion

Our EM maps and fits of Hsp104 provide a new working model that constitutes a major revision to the currently accepted model of ClpB/Hsp104 oligomer organization, in which the coiled-coil domain extends radially outwards from NBD1 (Lee et al., 2003, 2007). An overlay of the Hsp104 and ClpB maps is shown in Supplementary Figure 6. The radically different arrangement of the complex, with an expanded cavity and the coiled-coil domain intercalated between the AAA+ domains, is compatible with genetic and biochemical data and provides explanations and new ideas about the mechanism of disaggregation and allosteric interactions in this important class of molecular machines.

### The Coiled Coil Intervenes in All Interdomain Communication in Hsp104

The lack of density for most of the coiled-coil domain in the *T*ClpB EM maps, along with its variable orientation in the crystal structure, led to the suggestion that it is highly mobile in the hexamer (Lee et al., 2003, 2007). However, the density in our Hsp104 maps is sufficient to account for all domains, albeit with substantial hinge rotations, especially for the coiled coil. Moreover, the observed density is clearly incompatible with the classical hexameric packing of the AAA+ domains. Instead, it reveals that the coiled-coil domain intervenes to prevent normal packing by covering the cavity-facing side of the ATP binding groove of NBD1 at one end and intercalating between NBD1 and the adjacent NBD2 at the other end (Figure 2). There are two major functional consequences of this inserted position of the coiled coil. First, it prevents the expected intersubunit interactions, in particular involving the arginine finger contact. Second, it is in a position to participate in translocation of substrates. These two activities are discussed below.

### An Alternate Arginine Finger Mechanism Involving the Coiled-Coil Domain

The arginine finger interaction is based on the structure of the Ras GTPase bound to GDP-AIF_3_ and its cofactor GAP-334 (Scheffzek et al., 1997). The catalytic arginine side chain that stabilizes the transition state during GTP hydrolysis forms a close contact that completes the active site of the GTPase. Sequence alignments and mutational studies support an Arg finger mechanism for AAA+ proteins (Ogura et al., 2004), but the structure-function relations in many cases are unclear. For example, in various crystal structures of AAA+ oligomers, either nucleotide is absent or the predicted arginine finger contact is not made despite the presence of nucleotide analog (Bochtler et al., 2000; Massey et al., 2006). In other examples, mutation of R388 in NSF NBD1 leads to a loss of its biological activity, but does not abolish ATPase activity (Matveeva et al., 2002) and the SV40 large tumor antigen helicase employs three positively charged residues supplied in *trans* to the active site, one of which is a lysine (Gai et al., 2004).

Because the predicted arginine fingers in Hsp104 are rotated away from the ATP binding site in both NBDs when ATPγS is bound, and the coiled-coil domain prevents the classical arginine interaction in NBD1, we propose that the conserved arginine residue R419 and possibly additional charged residues on the coiled coil take over the catalytic role of the Arg-finger in NBD1. In support of this idea, mutation of R419 on the coiled coil greatly reduces ATPase activity and NM fiber disassembly activity but does not impair hexamerisation (Figure 6). Mutation of the predicted arginine finger R334 shows the same effects as the mutation of R419, but leads to a less stable Hsp104 oligomer. Thus, the role of R334 might be more related to optimal maintenance of oligomer contacts during the ATPase cycle. Interestingly ATP hydrolysis of Hsp104^R765M^, mutant of the predicted arginine finger in NBD2, is initially inhibited before recovering to wild-type activity. Despite the lack of a hexamerisation defect in crosslinking experiments (Figure 6), this effect could indicate defective intermolecular interactions that can be overcome once the hexamer is formed. The remarkably low ATPase activity of the R444M mutant might also be explained by a cooperativity defect in the hexamer, since its expression in a wild-type Hsp104 background greatly interferes with function (Figure 6F). Consistent with such roles, R765 and R444 are located at the intersubunit interface in our fit (Figure 5).

In summary, mutation of any of the conserved arginines in the coiled coil results in severe effects on ATPase activity, prion disaggregation and thermotolerance, which can be explained when the coiled-coil domain is intercalated between NBD1 and NBD2. On the other hand, it would be difficult to account for these findings, and in particular the effects of the R444M mutation, with an external placement of the coiled coil (Lee et al., 2003, 2007).

### Action of the Coiled-Coil Domain inside the Central Channel

Several studies provide experimental support for an interaction between the coiled-coil domain and NBD1. Point mutations in the helix 3 end of the coiled-coil domain reduce thermotolerance in *Arabidopsis* seedlings, disturb interactions of Hsp104/ClpB with cochaperones and substrates and strongly affect the ATPase cycle (Lee et al., 2005; Schirmer et al., 2004; Haslberger et al., 2007). Interestingly, mutations in the cavity facing surface of NBD1 can compensate for effects of the coiled-coil mutations in *At*Hsp101, pointing to a role of the coiled coil in modulating the ATPase activity of NBD1 and positioning the axial channel loops (Lee et al., 2005). Crosslinks in the helix 3-bearing end of the ClpB coiled coil that limit its flexibility impair the association of NBD1 with the coiled coil, suggesting that this interaction involves different conformational states of the sub-domains (Haslberger et al., 2007). The effects of the R495 point mutation (Figure 6) resemble previously reported results on helix L3 mutations, further stressing the importance of the interaction between NBD1 and the coiled coil. This body of biochemical and genetic data is well accounted for by our proposed arrangement of the Hsp104 hexamer.

A recent study on prion-specific activity of Hsp104 detected three mutations that resulted in loss of [*PSI*^+^] propagation while maintaining general thermotolerance (Kurahashi and Nakamura, 2007). In our model, L462R on helix L2 of the coiled coil alters a conserved leucine and introduces an arginine close to the ATP in NBD1 and D704N is located on NBD2 at the contact interface with the coiled coil (Figure 5). Moreover, we show that similarly positioned mutations of R444 and R765 severely impair NM fiber disassembly by Hsp104. These observations and the position of the apical end of the coiled coil inside the complex support the notion that the coiled coil plays a direct role in disaggregation within the cavity.

Hsp104 contains conserved, channel-facing tyrosine residues in both NBDs, and mutation of the one in NBD2 significantly reduces thermotolerance (Lum et al., 2004). The comparatively weak effect of the NBD1 Tyr mutation suggests that other regions of the protein are also important for disaggregation. Indeed, mutations of the conserved tyrosine residue on helix L3 abolish disaggregation activity in ClpB (Haslberger et al., 2007). We therefore propose that this residue contributes to substrate translocation through the central channel. The results of Haslberger et al. (2007) show that the coiled-coil mutants are functional in threading activity but not in the disaggregation of substrates that require the DnaK chaperone system. Our structure raises the possibility that the coiled coil provides extra force inside the cavity to tackle highly aggregated proteins or prions.

### Conclusions

The conformation that Hsp104 adopts in the hexamer differs considerably from those in the non-hexameric ClpB crystal structures. The observed differences are therefore due to intrinsic differences between ClpB and Hsp104 and/or reflect the effects of crystallization. Crystallization effects have been observed in other ATPases which exhibit different conformations in solution and in a crystal lattice, or in which different nucleotide states occupy the same conformation (e.g., see Xu et al., 1997; Ranson et al., 2006; Ditzel et al., 1998; Wang et al., 2001). However, the crystal structure could also represent the conformation of monomeric Hsp104, suggesting that a rearrangement of the coiled-coil domain could inhibit ATPase activity in the monomer and promotes disassembly in the absence of nucleotide and/ or substrate or during remodeling of intractable substrates.

The large cavity formed by the Hsp104 hexamer could allow for extraction of partially structured peptide chains and peptide loops, so that aggregates can be tackled in various ways. To fully understand the disaggregation mechanism a more detailed analysis of the conformational states in the hexamer is necessary.

## Experimental Procedures

### Mutagenesis, Protein Purification, and Biochemical Assays

The Hsp104^R334M^, Hsp104^R419M^, Hsp104^R444M^, Hsp104^R495M^ and Hsp104^R765M^ mutants were generated by QuikChange® mutagenesis (Stratagene). Hsp104^N728A^ and Hsp104 Δ1-157 (ΔN) were purified from *E.coli* BL21-Codon Plus®-RIL cells (Stratagene) as described (Hattendorf and Lindquist, 2002a). Hsp104, Hsp104^R334M^, Hsp104^R419M^, Hsp104^R444M^, Hsp104^R495M^, and Hsp104^R765M^ were purified as described (Shorter and Lindquist, 2004). To measure ATPase activity, wild-type or mutant Hsp104 proteins (0.2 μM monomer) were incubated at 25°C in 40 mM HEPES-KOH (pH 7.4), 150 mM KCl, 20 mM MgCl_2_, 10% glycerol, 1 mM DTT, and 1 mM ATP. The release of inorganic phosphate was determined using a malachite green phosphate detection kit (Innova). Hexamerization of wild-type or mutant Hsp104 proteins (0.2 μM monomer) was assessed by glutaraldehyde crosslinking as described (Parsell et al., 1994), except that Hsp104 was initially exchanged into 40 mM HEPES-KOH (pH 7.4), 150 mM KCl, 20 mM MgCl_2_, and 1 mM DTT. Prion disassembly was performed as described (Shorter and Lindquist, 2004).

### Thermotolerance Assays

W303a (*Mat*a, *can1-100, his3-11,15, leu2-3,112, trp1-1, ura3-1, ade2-1*) and the isogenic strain where the *HSP104* gene is deleted (W303a Δ*hsp104*) (Sanchez and Lindquist, 1990) were transformed with either a centromeric plasmid bearing the *HSP104* promoter, pHSE, or pHSE encoding Hsp104, Hsp104^R334M^, Hsp104^R419M^, Hsp104^R444M^, Hsp104^R495M^, or Hsp104^R765M^. Transformants were grown in SD-ura at 30°C to mid-log phase (5 × 10^6^ cells/ml), then either maintained at 30°C (untreated) or incubated at 37°C for 30 min (preconditioned). Cells were then incubated at 50°C for increasing times. Heat shock was terminated by transferring tubes to ice. Cells were then diluted in ice-cold SD-ura, and various dilutions were immediately plated onto SD-ura and allowed to recover for 3 days at 30°C to assess viability. Colonies were counted using an aCOLyte automated colony counter (Synbiosis). Immunoblot analysis showed that each Hsp104 protein was expressed at a similar level under equivalent conditions (data not shown).

### Cryo-Electron Microscopy

Proteins were diluted to a final concentration of 0.3 mg/ml in 20 mM HEPES (pH 7.5), 20 mM NaCl, 10 mM MgCl_2_, 1 mM DTT and 2 mM ATPγS. A 3.5 μl sample of the solution was applied to glow discharged, lacey carbon film on copper grids (300 mesh). After 30 s excess solution was blotted and the grid was flash frozen in liquid ethane. Cryo EM was carried out on a Tecnai F20 FEG operated at 200 kV under low dose conditions. Images were taken at a magnification of 50,000x with defocus ranging from 1.5–4.1 μm.

### Image Processing and 3D Reconstruction

Micrographs were digitized on a SCAI microdensitometer (Zeiss) at a sampling rate of 1.4 Å per pixel at the specimen level. A total of 9497 particles (Hsp104^N728A^) and 5353 particles (Hsp104 ΔN) were manually selected using the MRC program Ximdisp (Crowther et al., 1996). The defocus and astigmatism of the micrographs were determined with the MRC program CTFFIND2 and phases were corrected for effects of the contrast transfer function in SPIDER (Frank et al., 1996). Initial image processing was done with IMAGIC-5 (van Heel et al., 1996). Particle images were binned to 2.8 Å per pixel, band-pass filtered between 6 and 160 Å, normalized and centered by iteratively aligning them to their rotationally averaged sum. Initial class averages were obtained by three rounds of classification based on multivariate statistical analysis (MSA) followed by multi-reference alignment (MRA) using homogenous classes as new references. The symmetry of the complex was determined by extracting top views of Hsp104 ΔN, randomly rotating them and subjecting them to MSA (suppl. Figure 1A). Subsequently a low-resolution density map was created by angular reconstitution with 6-fold symmetry for each dataset. Particle orientations were refined in multiple cycles of MRA, MSA and angular reconstitution and the resulting 3-D reconstruction was used as an initial model for projection matching in SPIDER. Based on the cross correlation coefficient, ∼70% of the images were included in the 3-D reconstructions using the SIRT algorithm (Gilbert, 1972). After 13–16 cycles of projection matching ∼90% of the assigned angles were stable (suppl. Figure 1B). The final reconstructions comprised ∼4000 and ∼7000 particles for Hsp104 ΔN and Hsp104^N728A^ respectively, resulting in structures at 11 and 13 Å resolution, estimated by Fourier Shell Correlation with a 0.5 correlation cut-off and loose masking. Without masking, the resolutions were 14 and 15 Å for Hsp104 ΔN and Hsp104^N728A^ respectively (Figures S1C and S1D). Possible explanations for lower quality of the Hsp104^N728A^ map, despite inclusion of almost double the amount of data as in the Hsp104 ΔN map, are heterogeneity of the Hsp104^N728A^ dataset and/or deviations from the imposed symmetry. Our unpublished results support the latter explanation.

### Atomic Structure Fitting

The absolute hand of the Hsp104^N728A^ map was determined by mapping out the cross correlation coefficient (CCC) between image tilt pairs of negatively stained complexes for all possible tilt axes based on the prediction of orientations from the untilted particles (Rosenthal and Henderson, 2003). The CCC was better for the hand shown (Figure S3). The relative orientations of the ΔN and full-length Hsp104 maps were determined by comparing the CCCs for the two possible orientations in the UCSF Chimera package (Pettersen et al., 2004). The CCC for the alignment shown is 0.727, compared to 0.592 if one of the maps is flipped over.

Docking was done using an Hsp104 homology model based on known X-ray structures with pdb entry codes 1qvrA.pdb, 1qvrB.pdb, 1qvrC.pdb (ClpB, all 47.1% identity), 1ksf.pdb (ClpA NBD1, 53.05% identity), 1r6b.pdb (ClpA NBD1, 53.05% identity) and 1jbkA.pdb (ClpB NBD1, 63.3% identity) generated with SWISS-MODEL (Schwede et al., 2003). The AAA+, the coiled coil and the N-terminal domains were fitted manually as rigid bodies into the EM densities using PYMOL (www.pymol.org). Automated fitting in Chimera optimised the fit for NBD2. The fit for NBD1 in Hsp104 ΔN was optimised by docking the AAA+ subdomains individually. All figures were prepared using PYMOL, except for Figure 1, which was done with Chimera.

## Accession Numbers

The cryo-EM maps have been deposited in the 3D-EM database, EMBL-European Bioinformatics Institute (Cambridge) with the accession codes EMD-1358 (Hsp104 N728A) and EMD-1359 (Hsp104 ΔN).

## Supplemental Data

Document S1. Six Figures
