# Supplementary material for: Atypical AAA+ Subunit Packing Creates an Expanded Cavity for Disaggregation by the Protein-Remodeling Factor Hsp104
Source: Cell. 2007 Dec 28;131(7):1366–77. doi: 10.1016/j.cell.2007.10.047 (PMC2211523; doi:10.1016/j.cell.2007.10.047)
Supplement: Document S1. Six Figures [file mmc1.pdf]

Cell, Volume *131*

Supplemental Data

Atypical AAA+ Subunit Packing Creates an Expanded Cavity for Disaggregation by the Protein-Remodeling Factor Hsp104

Petra Wendler, James Shorter, Celia Plisson, Anil G Cashikar, Susan Lindquist, and Helen R Saibil

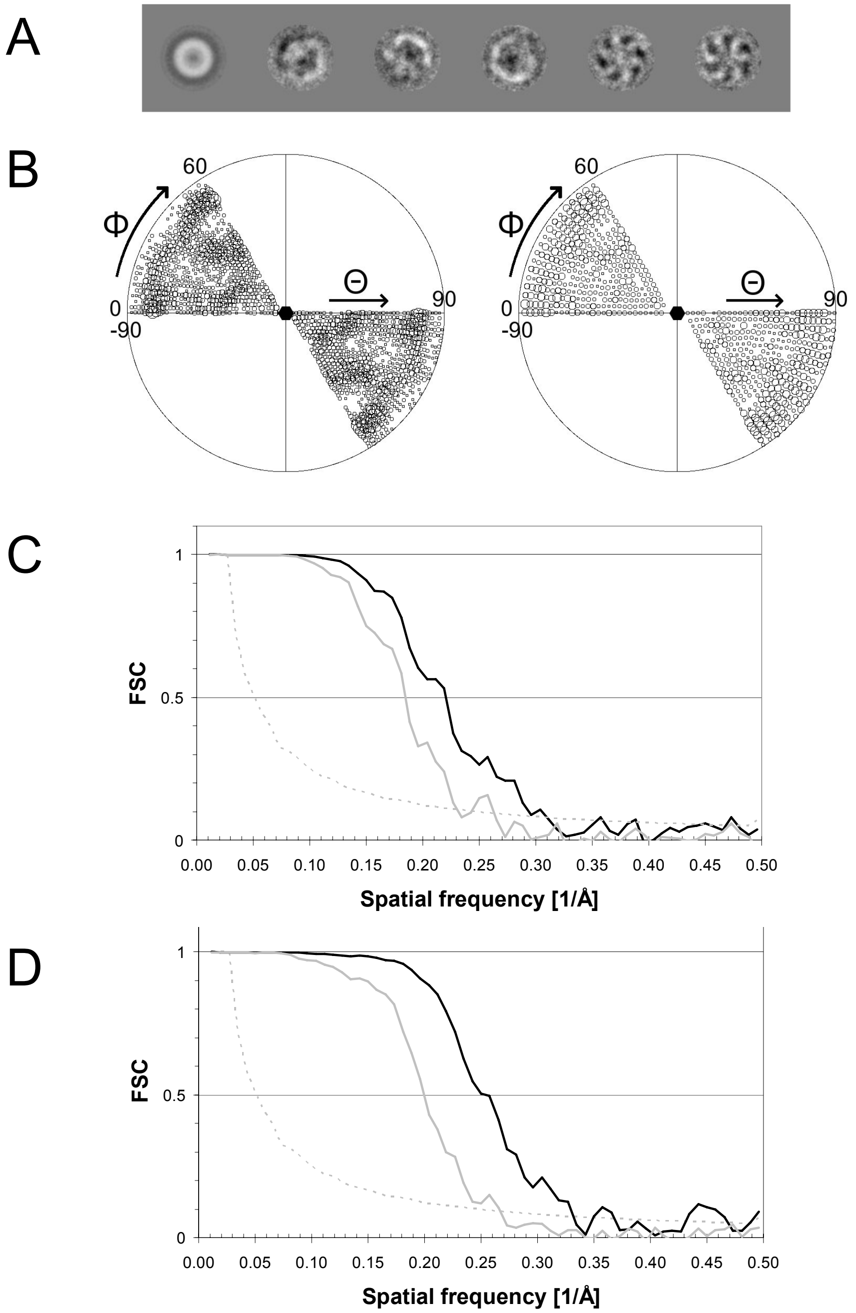

Figure S1. Symmetry analysis and map validation

(A) Symmetry analysis of Hsp104 ΔN. Eigen images derived from MSA of randomly rotated top views which were extracted from the dataset after two rounds of reference-free alignment in IMAGIC. The symmetry of Hsp104 ΔN is represented by the last two eigen images.

(B) Euler angle distribution for the Hsp104 full length (left) and ΔN (right) dataset after refinement with projection matching. Discrete directions with 2º (left) and 3º (right) azimuthal equidistant angular spacing are represented by a circle, whose size corresponds to the number of particles matching that direction. The position of the C6 symmetry axis and the directions of the polar angles (Θ) and azimuthal angles (Φ) are given.

(C) Fourier shell correlation curves of full length Hsp104 without mask (grey) and with loose masking (black). The resolution measured using the 0.5 cut-off criterion is 15.2 Å and 12.7 Å, respectively.

(D) Fourier shell correlation curves of ΔN Hsp104 without mask (grey) and with loose masking (black). The resolution measured using the 0.5 cut-off criterion is 14.0 Å and 11.1 Å, respectively.

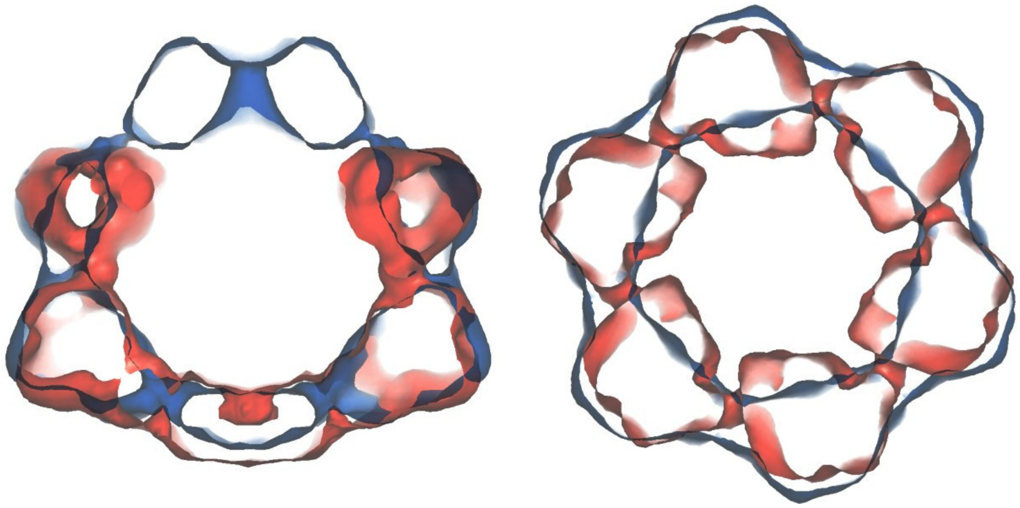

Figure S2. Comparison of full-length (blue) and ΔN Hsp104 (red) 3D reconstructions.

Left: 20 Å section through the overlaid structures in the side view. Right: Cross section of the overlaid structures through NBD1.

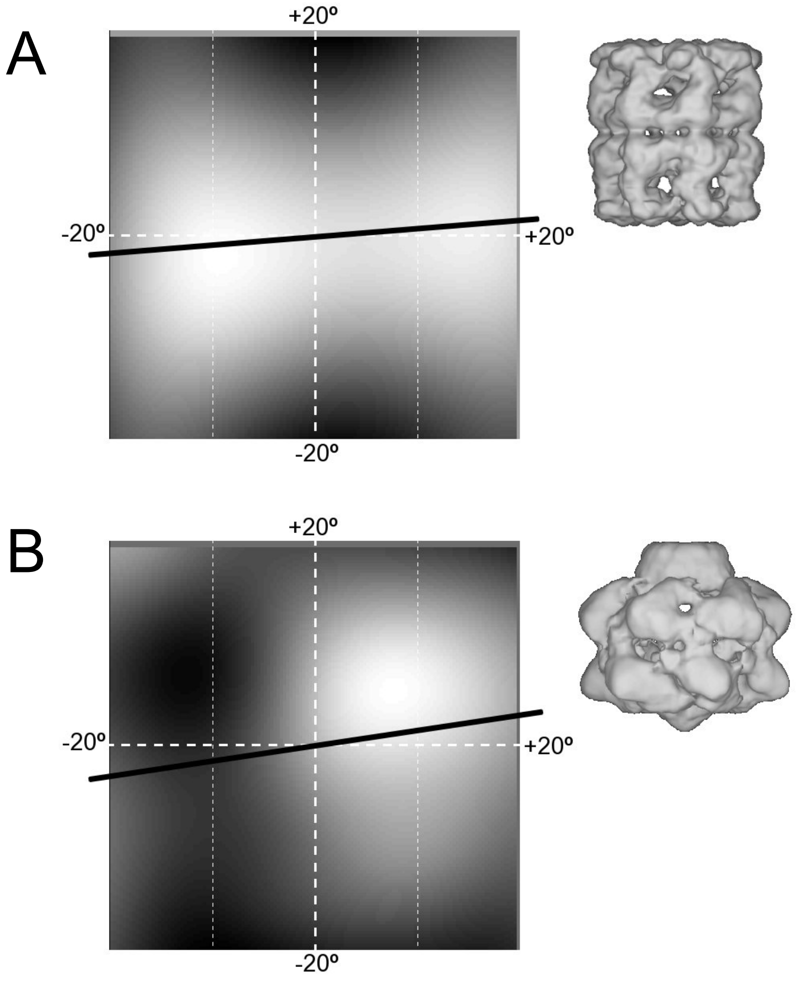

Figure S3. Tilt data for hand determination of maps.

Determination of the absolute hand of ATPγS bound GroEL (A) and Hsp104 (B) from negatively stained particles according to Rosenthal and Henderson, 2003 (Optimal determination of particle orientation, absolute hand, and contrast loss in single-particle electron cryomicroscopy. J Mol Biol *333*, 721- 745). Images show the average cross correlation coefficients for 99 and 100 particle images recorded at a tilt of -8.59° for GroEL and -13.17° for Hsp104 (as determined with CTFtilt). Cross correlation coefficients were determined in 1° increments for each tilt transformation up to 20° along the x and y axis and displayed as grey values. The direction of the known tilt axis of the microscope is shown as a black diagonal line. The orientations

of the untilted particles were determined by aligning them against 3D reconstructions of ATP bound GroEL and ATPγS bound Hsp104 respectively (shown on the right).

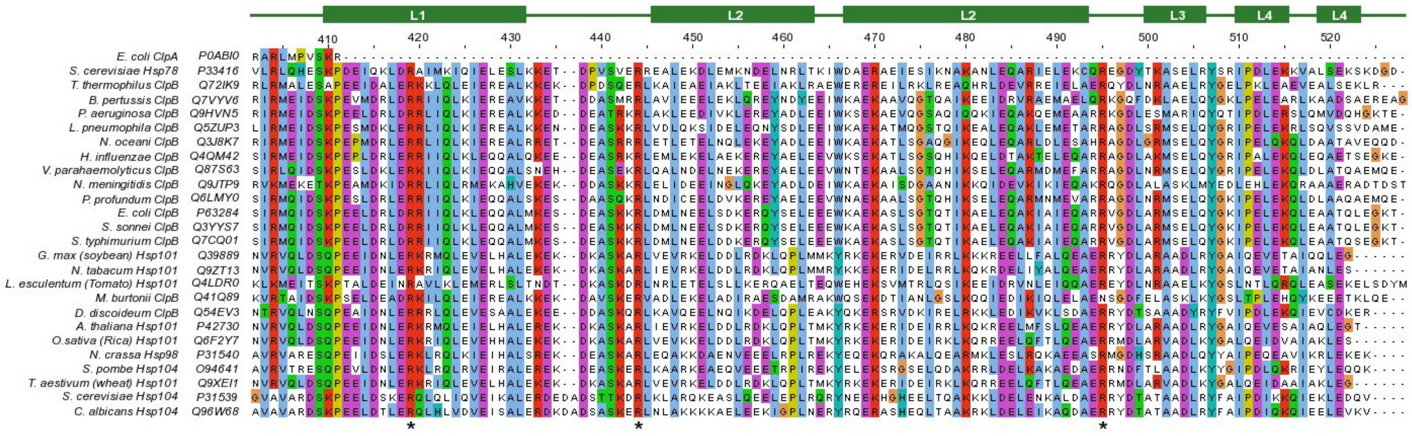

Figure S4. Multiple sequence alignment of Hsp100 proteins.

Sequence alignment of 26 Hsp100 proteins using ClustalW (Chenna, R., Sugawara, H., Koike, T., Lopez, R., Gibson, T.J., Higgins, D.G., and Thompson, J.D., 2003. Multiple sequence alignment with the Clustal series of programs, Nucl Acid Res *31*. 3497-3500.), visualised with JalView (Clamp, M., Cuff, J., Searle, S.M., and Barton, G.J., 2004. The Jalview Java alignment editor, Bioinformatics *20*. 426-427.). Alignment for the coiled-coil region is displayed in Clustal color code. Protein names, origin and UniProt entry codes are given. Conserved arginines R419, R444 and R495 are marked by *. Numbering is based on *S. cerevisiae* Hsp104. Secondary structure elements as identified in the Hsp104 homology model are presented as bars and labeled according to the ClpB crystal structure (Lee, S., Sowa, M.E., Watanabe, Y.H., Sigler, P.B., Chiu, W., Yoshida, M., and Tsai, F.T., 2003. The structure of ClpB: a molecular chaperone that rescues proteins from an aggregated state. Cell *115*, 229-240).

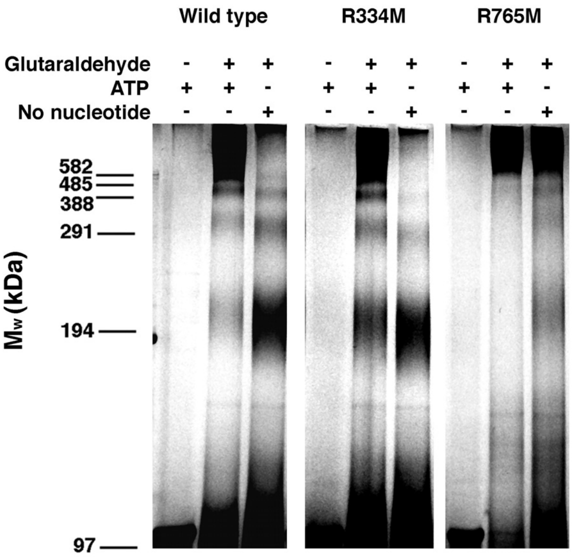

Figure S5. Cross-linking to examine oligomeric states of Hsp104 variants.

Wild type or mutant Hsp104 proteins (0.2 μM) in the presence or absence of ATP (1 mM) were either cross-linked with 0.1% glutaraldehyde for 10 min or left untreated. Cross-linking reactions were quenched by addition of 1 M glycine, and trichloroacetic acid-precipitated proteins were separated on 4% SDS-PAGE gels. The molecular weights of cross-linked species were estimated by comparison with cross-linked phosphorylase b.

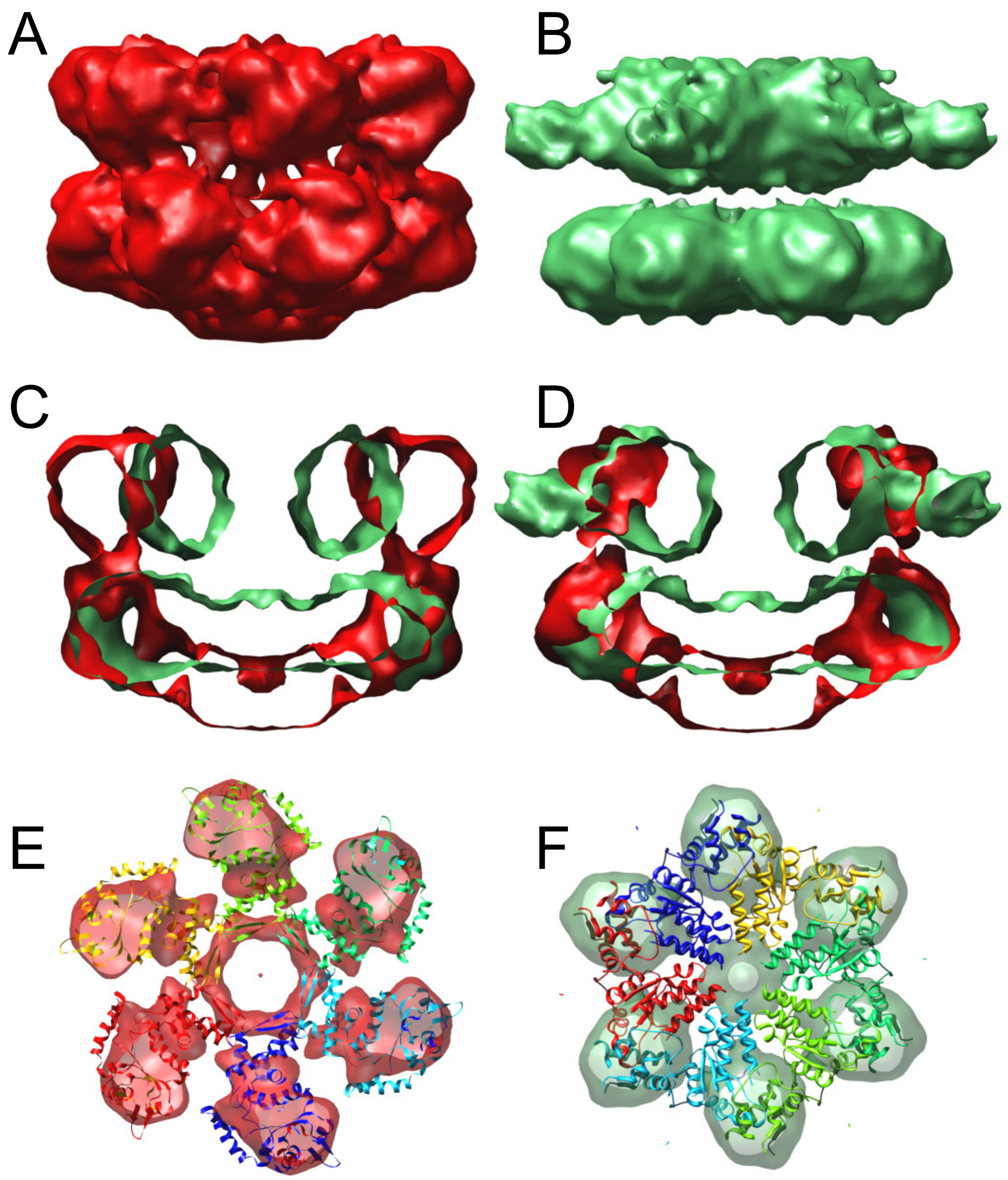

Figure S6. Comparison of cryo EM maps of Hsp104 and ClpB.

(A) Hsp104 ΔN and (B) the ClpB-AMPPNP map of Lee et al, 2007 (Lee, S., Choi, J.-M., and Tsai, F.T.F.. Visualizing the ATPase Cycle in a Protein Disaggregating Machine: Structural Basis for Substrate Binding by ClpB. Molecular Cell *25*, 261-271). Both maps are shown at thresholds enclosing the volume of all domains except

the N-terminus. In the ClpB cryo EM work, the oligomers were stabilized by glutaraldehyde cross linking. The maps were aligned by cross-correlation in Chimera and two section views are overlaid in C and D. The radial extensions from NBD1 in the Lee et al map were proposed to be the coiled-coil domains. (E) Cross-section through the lower ring (NBD2) of the Hsp104 contoured at high threshold to show the strongest density features, with the corresponding atomic structure fit. (F) High density section of the ClpB map, with the NBD2 ring of p97 (Huyton, T., Pye, V.E., Briggs, L.C., Flynn, T.C., Beuron, F., Kondo, H., Ma, J., Zhang, X., and Freemont, P.S., 2003. The crystal structure of murine p97/VCP at 3.6 A. Journal of Structural Biology *144*, 337-348) superposed to show the fit of a canonical AAA+ ring.
